# Effects of Iodixanol on Respiratory Functions during Coronary Angiography and the Role of Body Composition

**DOI:** 10.1155/2018/2140219

**Published:** 2018-06-20

**Authors:** Derya Guzel, Mustafa Gokhan Vural, Ramazan Akdemir

**Affiliations:** ^1^Department of Physiology, School of Medicine, Sakarya University, Sakarya, Turkey; ^2^Department of Cardiology, School of Medicine, Sakarya University, Sakarya, Turkey

## Abstract

**Purpose:**

The purposes of this study are to assess the acute effects of iodixanol, an iso-osmolar contrast media, on pulmonary functions and to evaluate the body composition in order to find out its role in causing this deterioration.

**Methods:**

35 male and 25 female patients undergoing diagnostic coronary angiography (CA) were enrolled in the study. Before CA, all patients' body compositions were evaluated by measuring their body mass indexes (BMIs) and waist-to-hip ratios (WHRs). Total body waters (TBWs), fat masses (FMs), fat-free masses (FFMs), and basal metabolism rates (BMRs) were measured via bioimpedance analysis. The CA was performed via radial artery route using iodixanol in every patient. The pulmonary function tests of these patients were performed before, during, and 2 hours after the CA. FEV1∆, FEF_25–75%_∆, and FVC∆ parameters were calculated by subtracting the measured baseline value from the measurement after the CA.

**Results:**

Angiography caused significant reduction in forced expiratory volume in 1 sec (FEV1, from 94.17 ± 18.83 to 84.45 ± 18.31, *p* < 0.0001), forced vital capacity (FVC, from 96.57 ± 15.82 to 88.31 ± 17.96, *p* < 0.0001), and forced expiratory flow at 25–75% (FEF_25–75%_ from 82.54 ± 24.26 to 72.11 ± 25.41, *p*=0.001) and remained lower after 2 h after CA in male patients, respectively. FEV1 values were 103.40 ± 17.79 to 94.96 ± 17.063 (*p*=0.004); FVC values were 107.20 ± 19.03 to 99.08 ± 20.56 (*p*=0.009); and FEF_25–75%_ values were 83.92 ± 24.30 to 73.24 ± 20.45 (*p*=0.005) before and after CA and remained lower after 2 h after CA in female patients, respectively. FEV1/FVC ratio remained unchanged. FEF_25–75%_∆ was statistically correlated with FFM, TBW, and WHR (*p* < 0.05; *r*=−0.344, *r*=−0.347, and *r*=0.357, resp.), and FVC∆ was correlated with WHR in male patients (*p*=0.018, *r*=397).

**Conclusions:**

Our data suggested that diagnostic CA using iodixanol, an iso-osmolar contrast media, leads significant impairment in respiratory functions. Due to the persistence of these reductions even 2 hours after CA, ventilatory functions should be considered especially in patients whose body compositions or hydration levels are not within the desired physiological range.

## 1. Introduction

Coronary angiography still remains a gold standard diagnostic tool for the definite diagnosis of coronary artery disease. Although the diagnostic accuracy and the tolerance of the currently available contrast agents are high, their administration may cause acute complications in patients with increased risks. Moreover, interventional cardiology patients, such as acute myocardial infarction patients, may already be subject to a higher complication risk due to the underlying conditions, and the contrast agent that is used may even have cardiac adverse effects and complications [1]. Contrast media-related complications, as well as the cardiovascular problems, are among the causes for hospitalizing the patients and related financial burdens [2, 3].

Given that the number of the types of the interventional cardiology procedures has been increasing over the past few years, the search for a better-tolerated agent continues to eliminate the risks associated with contrast agents. Iso-osmolar contrast agent is one of the nonionic iodinated contrast agent types, in line with the evidence that it has fewer side effects than low-osmolar contrast media [4].

Among those complications, contrast-induced nephropathy is studied in the majority of published literature before. Adverse effects of angiographic contrast agents diminished dramatically by clinical precautions, premedications before the angiography, reducing the amount of contrast agent usage, and also increasing the usage of iso-osmolar nonionic angiographic contrast agents during angiography procedures.

Respiratory system, while it works together with the cardiovascular system for oxygen homeostasis, can be directly affected by cardiovascular disorders [5] or indirectly by diagnostic contrast agents. There are scarce data on the adverse effects of angiographic contrast agents on respiratory functions. Although vasovagal and chemotoxic-organ-specific side effects [6] of contrast agent are evaluated, there are a little data about the respiratory effects associated to those contrast agents. We have thought that osmolality does matter in respiratory functions during CA and an iso-osmolar contrast agent might be safe. Furthermore, body composition might have a role in respiratory functional changes during CA using a contrast agent. We aimed at evaluating the acute effects of iodixanol, an iso-osmolar contrast agent, on pulmonary functions and at evaluating the body composition regarding the question of whether it plays a potential role in causing this deterioration.

## 2. Methods

### 2.1. Study Design

A study group was chosen among the patients who had been planned diagnostic CA: sixty of 95 consecutive patients undergoing diagnostic coronary angiography. 35 male and 25 female patients who fulfil the inclusion and exclusion criteria were enrolled in the study. Before CA, all patients' body compositions were evaluated by measuring their body mass indexes (BMIs) and waist-to-hip ratios (WHRs). Total body waters (TBWs), fat masses (FMs), fat-free masses (FFMs), and basal metabolism rates (BMRs) were measured via bioimpedance analysis. The CA was performed via radial artery route by the standard technique as three projections for the left system coronary arteries and two projections for the right coronary artery using iodixanol in every patient.

Participants with clinically significant respiratory disease (asthma, chronic obstructive or restrictive pulmonary disease), anormal pulmonary function test results before CA (FVC% < 80%, FEV1 < 80%, FEV1/FVC < 75%), cardiovascular disease (myocardial infarction, previous coronary intervention or surgery, and peripheral arterial disease), renal insufficiency (estimated glomerular filtration rate <60 ml/min), and previous allergic reactions to contrast-agent exposure were not enrolled in the study. Patients with critical coronary lesions on coronary angiography were excluded from the study. And also patients who requiring left ventriculography, total contrast agent volume exceeding 60 cc, difficulty to engage coronary ostium by diagnostic catheters or exchanging from radial route to femoral route, and failure of radial artery cannulation during CA were excluded the study.

Further exclusion criteria for the study were as follows: moderate-to-severe valvular heart disease, history of pulmonary embolism, pulmonary hypertension (mean pulmonary artery pressure >40 mmHg on echocardiography), abnormal serum electrolyte values, and abnormal thyroid function. A written consent was obtained from all the patients, and our local ethical committee approved the study.

### 2.2. Assessment and Measures

Body compositions were evaluated using the bioelectrical impedance method (TBF-300® device; Tanita, Illinois, USA). Body mass indexes (BMIs), basal metabolism rates (BMRs), body fat masses (BFMs), fat-free masses (FFMs), and total body waters (TBWs) of the patients were measured. The waist-to-hip ratios (WHRs) were obtained by dividing the waist circumference to the hip circumference.

Pulmonary function tests (PFTs) were performed in accordance with the American Thoracic Society (ATS) criteria, using a pulmonary function analyser (MIR Spirolab III, Oberthulba, Germany). Individuals were informed before the measurements. First test was performed in a comfortable sitting position followed by a 15 min of resting. A mouthpiece was placed between the teeth and lips. Then, a strong expiration was performed by the patient followed by a deep inspiration after breathing 3 times in a relaxed way. The test was repeated 3 times to obtain the best values. FVC, FEV1, FEV1/FVC, forced expiratory flow between 25% and 75% of FVC (FEF_25–75%_), FVC% (expected), FEV1% (expected), FEV1/FVC% (expected), and FEF_25–75%_ (expected) were also measured. Then, coronary angiography routine procedures were performed through radial artery route by a single experienced angiographer with catheter exchange using 5 F Judkins diagnostic catheters over a 0.035-inch guide wire. Totally, six multiple-angled views of the left and right coronary arteries were recorded in all patients after hand injection. The contrast agent studied was iodixanol, an iodine-containing nonionic radiocontrast agent with a molecular weight of 1550.20 and iodine content of 49.1. The concentration used for the study was a commercially available formulation (Visipaque-320) containing osmolality of 290 mOsm/kg of water. All respiratory measurements were repeated immediately after CA and 2 hours after CA. FEV1∆, FEF_25–75%_∆, and FVC∆ parameters were calculated by subtracting the measured baseline value from the measurement after CA.

### 2.3. Statistical Analysis

Final data were evaluated by using the statistical package programme (SPSS 15 Chicago Inc.). Chi-square test or Fisher's exact test was used to evaluate categorical data. Whether continuous data had normal distribution or not, Shapiro–Wilks normality test was used. Independent *t*-test was used to compare differences between groups for continuous data. Paired samples' *t*-test was used to determine the difference between pretest and posttest. All the categorical data showed with frequencies and percentages, and all continuous variables showed mean ± standard division (mean ± SD) as descriptive statistics. Statistical boundary was given as 0.05. SPSS software version 20.0 for Windows (SPSS Inc., Chicago, IL, USA) was used for all statistical analyses. All data were tested for normality, and the appropriate nonparametric or parametric statistics were used. The numeric variables were compared using Wilcoxon rank-sum test. Any *p* value less than 0.05 was accepted as significant.

## 3. Results

Twenty-five female (56.44 ± 8.85) and 35 male patients (57.85 ± 11.91) were enrolled in the study. Characteristics of the patients are summarized in Table 1. The mean of the used contrast media was 40 ml for each patient.

A significant fall in FEV1% (from 94.17 ± 18.83 to 84.45 ± 18.31, *p* < 0.0001), FVC% (from 96.57 ± 15.82 to 88.31 ± 17.96, *p* < 0.0001), and FEF_25–75%_ (from 82.54 ± 24.26 to 72.11 ± 25.41, *p*=0.001) were seen after CA and insisted 2 h after CA in male patients. FEV1% (from 103.40 ± 17.79 to 94.96 ± 17.063, *p*:0.004), FVC% (from 107.20 ± 19.03 to 99.08 ± 20.56, *p*=0.009), and FEF_25–75%_ (from 83.92 ± 24.30 to 73.24 ± 20.45, *p*=0.005) were significantly decreased and insisted 2 h after CA in female patients (*p* < 0.01, *p* < 0.05, and *p* < 0.01, resp.; Table 2). FVC% values of 12 male and 6 female patients and FEV1% values of 18 male and 3 female patients were below 80% after CA. FVC% values of 10 male and 4 female patients and FEV1% values of 16 male and 2 female patients were below 80% 2 h after CA. Only three male patients' FEV1/FVC values were below 75% (8.6% of men) after CA. However, FEV1/FVC ratio remained unchanged after CA (*p*=0.237) and 2 h after CA (*p*=0.341) in patients.

In female patients, body mass index was 34.12 ± 6.91, BMR was 6172.40 ± 823.53, BFM_%_ was 40.68 ± 5.15, BFM_kg_ was 34.10 ± 11.52, FFM was 47.56 ± 6.74, TBW was 34.93 ± 4.99, and WHR was 0.85 ± 0.05. In male patients, body mass index was 28.72 ± 6.12, BMR was 7126.06 ± 1035.63, BFM_%_ was 24.88 ± 8.23, BFM_kg_ was 21.80 ± 10.74, FFM was 61.08 ± 9.06, TBW was 44.49 ± 7.54, and WHR was 0.96 ± 0.06. FVC∆ was correlated with WHR in male patients (*p*=0.018, *r*=397). FEF_25–75%_∆ was statistically correlated with FFM, TBW, and WHR in male patients (*p* < 0.05; *r*=−0.344, *r*=−0.347, and *r*=0.357, resp.; Table 3).

## 4. Discussion

The present study demonstrated acute restrictive alterations in respiratory functions in patients undergoing CA using iso-osmolar contrast agents, and it remains altered at least 2 hours after CA. Furthermore, we revealed that respiratory functions altered in obese and dehydrated or malnutrated patients. This observation might be a clue for a potential mechanistic relationship between metabolic or volume status and respiratory adverse effects of iso-osmolar contrast agents used in high-risk patients.

CA remains the gold standard imaging modality to diagnose coronary artery disease. In contrary to the wide usage in clinical practice, CA has some limitations inherently due to its invasive nature and the usage of iodine-based contrast media. Historically, first-employed contrast media agents were high-osmolar compared to blood osmolality and had been accused for severe reactions including acute renal failure, cardiovascular collapse, hemorrhage, atherosclerosis, and death. It was well documented that high-osmolar contrast agents caused acute plasma expansion and released vasoactive substances [7]. Second-generation nonionic low-osmolar or iso-osmolar contrast media agents partly overcame these problems and caused fewer contrast-induced adverse events. It was shown that second-generation contrast media agents cause also hemodynamic alterations transiently in contrast to hyperosmolar agents. The potential mechanism of these acute alterations was suggested that rising in cardiac output and alteration in blood cells rheology is the major reason rather than pulmonary vascular reactivity.

The contrast agent can cause adverse respiratory reactions such as overt bronchospasm, laryngeal oedema, cardiac failure and loss of consciousness, and [8] pulmonary oedema. These severe adverse reactions are rare, unpredictable, and potentially fatal. Therefore, they must be recognized and treated promptly to prevent permanent morbidity or death [9]. Pulmonary function tests are among the beneficial methods to evaluate respiratory changes of patients. Lungs, chest wall, and respiratory muscles play a significant role in the determination of respiratory functions. If the results of these tests indicate a restrictive pattern, it can be interpreted that the respiratory disorders may have originated paranchim and so on; if these results indicate an obstrictive pattern, the disorders may have originated airway pathologies like broncoconstriction and so on [10]. According to our results, angiography caused significant reduction in FEV1, FVC, and FEF_25–75%_ values, and FEV1/FVC ratio was unchained. These reductions are in favour of asymptomatic restrictive pattern in pulmonary functions. Ozhan et al. [11] reported in their study that diagnostic CA using iohexol, a hypo-osmolar contrast agent, decreases ventilatory functions in a small but significant extent in patients without any overt pulmonary disease. They have suggested that contrast agent during CA increases the pulmonary artery pressure possibly through several mechanisms: a direct action on vascular smooth muscle, the release of endogenous vasoactive mediators (such as endothelin, histamine, serotonin, and nitric oxide), neural reflexes, and rheological properties of the red blood cells.

According to the literature, it was suggested that released mediators may cause this condition. In the study of Simon et al., it was demonstrated that plasma histamine, complement haemolytic activity, and fibrin split products were changed in patients undergoing radiographic contrast media infusions [12]. Mast cells and basophiles are mainly responsible for mediator release, which may cause disorders. Afterwards, the Fc*ɛ*R and the anaphylatoxin receptors (C5aR and C3aR), which bind with IgE, C5a, and C3a, respectively, the coactivation of the coagulation, and the kinin-kallikrein systems participate in the inflammation cascade [13]. These systems work together, and they can cause adverse effects.

Although these factors cause the main adverse effects, studies demonstrated that these mediators' changes and clinical features hardly correlate [12]. Therefore, other modulating factors may play significant roles in determining whether a reaction will occur or not. For example, body composition may also be another important factor for evaluating the side effects; TBW can be a cheap, noninvasive indicator to evaluate hydration; and also, respiratory functions can rather be affected by fat mass, fat-free mass, and so on[14, 15]; thus, we hypothesized that these factors helps us to evaluate the respiratory performance. We investigated whether the total body composition can be a clue to prevent the respiratory adverse effects, and our study proved that, especially in male patients, FFM, WHR, and TBW were statistically correlated with FEF_25–75%_∆, and FVC∆ was correlated with WHR. Our findings can explain the underlying mechanisms for the development of contrast agents in particular conditions such as the reduced effective intravascular volume or severe dehydration [16]. And also, decreasing the incidence of renal failure induced by contrast agent's hydration can be explained by our findings that higher TBW may decrease the adverse effects of contrast agents.

Despite some investigations that evaluated the effects of nonionic iso-osmolar contrast agents in respiratory functions, our study was the first study that designed to find out acute and short-term respiratory alterations using hypo-osmolar contrast media agents during CA. Furthermore, we suggested that body composition might be determinant of respiratory alterations by altering pharmacokinetic properties of contrast media agents, and we measured body composition parameters before CA and compared respiratory parameters in a time-course manner.

Body composition might be a determinant of contrast media agent-induced pulmonary impairment. Total body water arises an easily available marker to get an insight into volume status. It was well documented that dehydration is the single preventable predictor of contrast-induced adverse effects. Reduction of effective intravascular volume or severe dehydration enables renal tubulointerstitial damage after high-volume contrast exposure in high-risk patients [17]. Many randomized clinical trial data suggested that hydration prior to contrast exposure minimizes or prevents contrast-induced renal damage and consequent systemic inflammatory cascade. For this reason, it is logical that estimation of high-risk patients beyond conventional risk factors can decrease contrast-induced morbidity and mortality. Furthermore, we showed that metabolic status indicated by body fat mass can take a part in the pathogenesis of contrast-induced side effects. Further experimental and large-scale observational studies are needed to elucidate this concept.

The major limitations of our study are as follows: (i) Partial oxygen saturation was not measured by blood gas analysis to determine whether contrast-induced restrictive pulmonary impairment caused subclinical hypoxemia. (ii) The study patients were not followed beyond 2 hours. Therefore, it cannot be concluded that acute alteration in respiratory function had an impact on procedural morbidity or mortality. For this reason, we cannot speculate about cost-effectiveness of screening consecutive patients. (iii) This study indicated a significant association between acute respiratory alteration and fat body mass. Nevertheless, it cannot be a cause-effect relationship.

## 5. Conclusion

Our study demonstrated that diagnostic CA using iodixanol, the iso-osmolar contrast media, causes significant reduction in FEV1, FVC, and FEF_25–75%_ in patients without any overt pulmonary disease. These alternations were maintained at each three values after 2 hours following the CA. Ventilatory functions should be considered especially in patients whose body composition or hydration is not within the desired physiological range.

## Figures and Tables

**Table 1:** General characteristics of patients.

Patient characteristics	Female (*n*=25), *n* (%)	Male (*n*=35), *n* (%)
Smoking status		
Smoker	9 (36)	14 (40)
<30 pack-years	6 (66.6)	6 (42)
≥30 pack-years	3 (33.4)	8 (58)
Nonsmoker	12 (48)	15 (42)
Ex-smoker	4 (16)	6 (17)

Systemic disorders		
None	10 (40)	20 (57.1)
Hypertension	11 (44)	9 (25.7)
Diabetes mellitus	2 (8)	5 (14.3)
Others	2 (8)	1 (2.9)

Current medications		
None	8 (32)	20 (57.1)
Βeta-blockers	6 (24)	1 (2.9)
Calcium-channel blockers	6 (24)	8 (22.9)
AT-II type receptor blockers	2 (8)	2 (5.7)
Others	3 (12)	4 (11.5)

**Table 2:** Alternations in respiratory functions during CA.

Pulmonary function test	Before CA		At the end of CA		2 hours after CA	
	Female	Male	Female	Male	Female	Male
FEV1%	103.40 ± 17.79	94.17 ± 18.83	94.96 ± 17.063^#^	84.45 ± 18.31^##^	95.20 ± 18.51^#^	84.25 ± 19.68^##^
FEV1 (L)	2.21 ± 0.42	2.89 ± 0.87	1.96 ± 0.48^##^	2.60 ± 0.68^#^	2.03 ± 0.48^#^:	2.65 ± 0.77^#^
FVC%	107.20 ± 19.03	96.57 ± 15.82	99.08 ± 20.56^#^	88.31 ± 17.96^##^	99.44 ± 19.69^*∗*^	88.71 ± 17.82^##^
FVC (L)	2.73 ± 0.57	3.76 ± 0.97	2.45 ± 0.65^##^	3.39 ± 0.92^##^	2.53 ± 0.64^#^	3.52 ± 1.01^#^
FEF_25–75%_	83.92 ± 24.30	82.54 ± 24.26	73.24 ± 20.45^#^	72.11 ± 25.41^##^	74.48 ± 24.74^#^	71.85 ± 26.71^##^
FEF_25–75%_ (L/s)	2.43 ± 0.54	2.81 ± 0.99	2.04 ± 0.63^#^	2.45 ± 0.88^#^	2.16 ± 0.67^*∗*^	2.36 ± 0.91^#^

Pulmonary function tests were statically lower at the end of CA and 2 hours after CA. ^*∗*^*p* < 0.05; ^#^*p* < 0.01; ^##^*p* ≤ 0.001. FEV1: forced expiratory volume in 1 s; FVC: forced vital capacity; FEF_25–75%_: forced expiratory flow between 25% and 75% of FVC.

**Table 3:** The comparisons of pulmonary function changes and body composition.

Patients	BMI (mean ± SD)	BMR (mean ± SD)	BFM (%) (mean ± SD)	BFM (kg) (mean ± SD)	FFM (mean ± SD)	TBW (mean ± SD)	WHR (mean ± SD)
Female (*n*=25)	34.12 ± 6.91	6172.40 ± 823.53	40.68 ± 5.15	34.10 ± 11.52	47.56 ± 6.74	34.93 ± 4.99	0.85 ± 0.05
Male (*n*=35)	28.72 ± 6.12	7126.06 ± 1035.63	24.88 ± 8.23	21.80 ± 10.74	61.08 ± 9.06^*∗*^	44.49 ± 7.54^*∗*^	0.96 ± 0.06^*∗*^

FEF_25–75%_∆ was statistically correlated with FFM, TBW, and WHR in men. FVC∆ was correlated with WHR. BMI: body mass index; BMR: body metabolism rate; BFM: body fat mass; FFM: fat-free mass; TBW: total body water; WHR: waist-to-hip ratio. ^*∗*^*p* < 0.05.

## Data Availability

The data used to support the findings of this study are available from the corresponding author upon request.
